# Editorial: Clinical trials in cardiovascular medicine: 2025—addressing residual cardiovascular risks through precision-guided approaches

**DOI:** 10.3389/fcvm.2026.1904928

**Published:** 2026-07-02

**Authors:** Euscia Y. Wang, Keman Xu, Ying Shao, Yifan Lu, Shih-Yu Hung, Bo Yuan, Haiyan Xiang, Hong Wang, Xiaofeng Yang

**Affiliations:** 1 University of California San Diego, La Jolla, CA, United States; 2 Lemole Center for Integrated Lymphatics and Vascular Research, Lewis Katz School of Medicine at Temple University, Philadelphia, PA, United States; 3 Department of Cardiovascular Sciences, Metabolic Disease Research and Thrombosis Research Center, Lewis Katz School of Medicine at Temple University, Philadelphia, PA, United States

**Keywords:** cardiovascular diseases, clinical trials, precision-guided approaches, residual cardiovascular risks, therapy

Cardiovascular disease is the leading cause of morbidity and mortality worldwide (1) and its prevalence increases with population aging. Coronary heart disease (CHD) has ranked first in disease burden despite substantial advances in pharmacotherapy, interventional cardiology, and preventive medicine. Major risk factors for CHD include age, sex, family history, hypercholesterolemia, hypertension, diabetes, obesity, and lifestyle. Pathologically, metabolism disorder and dysregulated renin-angiotensin-aldosterone system and autonomous nervous system contribute to the development of CHD. Inflammation, a convergent response of various risk factors, is involved in all stages of CHD pathogenesis, including atherosclerosis, unstable angina, myocardial infarction, cardiac remodeling, and heart failure (2, 3). Development of new treatments, particularly those that target residual risk beyond cholesterol-lowering, has been of substantial interest recently. These include clinical development of anti-inflammatory modalities, including blockers of interleukin 1β (4) and interleukin-6 (5), and colchicine (6, 7), which is the only approved drug for CHD, but with efficacy concerns (7). Others include triglyceride lowering agents (8), and lipoprotein (a) lowering agents (9). Recent discovery of sodium-glucose cotransporter 2 inhibitors (10, 11) and glucagon-like peptide-1 (GLP1) agonists (12, 13) for cardiovascular benefits in diabetes patients fuels clinical endeavor of exploring these diabetes drugs for further cardiovascular benefits in non-diabetic patients. Meanwhile, advances in biomarker science, multimodality imaging, and systems biology are enabling investigators to target new mechanisms and those previously difficult to quantify, while allowing risk stratification and personalized treatment to improve cardiovascular outcome. The integration of pharmacological interventions, biomarkers, multi-omics technologies, advanced imaging, and digital health tools is therefore reshaping the landscape of translational cardiovascular research.

This special issue on Clinical Trials in Cardiovascular Medicine 2025 published eleven clinical studies as summarized in Table 1.

**Table 1:** Summary of the 11 articles published in this research topic illustrating the contemporary cardiovascular clinical investigations.

First author	Article title	Key findings	Paper category	PubMed ID
Yaxing Wang	Protocol for a randomized controlled trial of Xueshuantong injection on myocardial injury and residual cardiovascular risk in patients with unstable angina	This exploratory clinical trial is designed to evaluate the efficacy and safety of Xueshuantong, an injection formulation derived from *Panax notoginseng* saponins, in reducing biomarkers of myocardial injury and residual inflammation in patients with unstable angina. This study may call for future large-scale confirmatory trials on disease progression and long-term prognosis.	Study Protocol	41195123
Ji Yong Jang	Efficacy and safety of mono antiplatelet therapy with colchicine in acute coronary syndrome patients following percutaneous coronary intervention: rationale and design of the MACT II trial	There is a growing clinical interest in minimizing bleeding complications while simultaneously addressing residual inflammatory risk in patients with acute coronary syndrome (ACS). By combining colchicine with aspirin-free mono antiplatelet therapy, the MACT (Mono Antiplatelet and Colchicine Therapy) II trial is an investigator-initiated, prospective, multicenter, single-arm study designed to evaluate the safety and efficacy of an aspirin-free, inflammation-guided treatment strategy in 490 patients with ACS undergoing percutaneous coronary intervention. This study explores a more individualized post-ACS treatment strategy that may help refine future secondary prevention paradigms.	Study Protocol	41221450
Baraa Shebli	Assessing the cardiovascular and potassium lowering effects of levalbuterol compared to albuterol: a randomized control trial	This randomized trial directly compares the cardiovascular profile and potassium-lowering efficacy of levalbuterol versus albuterol in hyperkalemic patients. Both medications showed similar patterns in heart rate changes, potassium levels, and blood pressure variations at different time points. This study has implications for optimizing treatment strategies and minimizing adverse events in hyperkalemic patients.	Clinical Trial	41437987
Yaru Ge	Effects of ginseng berry saponins on cardiorespiratory fitness in patients with SCAD: a randomized, double-blinded, placebo-controlled trial	In a randomized placebo-controlled trial of 100 patients with stable coronary artery disease, Zhenyuan capsule, a Chinese medicine consisting of ginseng berry saponins, significantly improves cardiorespiratory fitness. Serum proteomics analysis suggests potential involvement of IGF2, which is associated with cholesterol metabolism and the PI3K-Akt pathway. This study raises the possibility that ginseng berry saponins may be used as an adjunctive strategy to enhance cardiorespiratory rehabilitation beyond current pharmacotherapy.	Clinical Trial	41660510
Lise Mateo	Both acute and chronic caffeine consumption affect cardiovascular responses to total sleep deprivation	This randomized, placebo-controlled, cross-over clinical trial evaluated the effect of caffeine on the cardiovascular and inflammatory responses associated with sleep deprivation. The study highlights that acute caffeine intake provokes immuno-inflammatory and cardiovascular responses, and that chronic caffeine consumption should be limited to the lowest efficient doses in sleep-restricted populations.	Clinical Trial	41694313
Tong Li	Integrative multi-omics profiling of mild cognitive impairment in older patients combined with coronary heart disease: a cross-sectional cohort study	A cross-sectional study was designed to profile disease phenomics signature of mild cognitive impairment in elderly patients with coronary heart disease, through comprehensive multi-omics analyses. This study highlights the growing intersection between cardiovascular disease and cognitive impairment through an integrative multi-omics approach. By identifying molecular and metabolic signatures associated with mild cognitive impairment in elderly patients with coronary heart disease, the work supports the emerging concept of cardio-cerebral interaction and may contribute to future precision risk stratification strategies in aging cardiovascular populations. This study might provide a scientific foundation for early detection and intervention for cognitive impairment in the elderly population with coronary heart disease.	Study Protocol	41808745
XuqiangWei	Electroacupuncture for slow flow/no-reflow in patients with acute myocardial infarction undergoing percutaneous coronary intervention: a pilot randomized controlled trial	In a single-center, randomized, assessor-blinded pilot trial of 60 patients with acute myocardial infarction undergoing percutaneous coronary intervention, Intraoperative electroacupuncture was associated with reduced slow flow/no-reflow and attenuated early inflammation. This preliminary study demonstrates the feasibility of electroacupuncture as a non-pharmacological adjunctive strategy for addressing the persistent clinical challenge of slow flow/no-reflow during reperfusion treatment in acute myocardial infarction.	Clinical Trial	41890868
Ajay Gupta	A sham-controlled randomised trial evaluating the safety, acceptability, and efficacy of autonomic neuromodulation using transcutaneous vagal sensory stimulation in uncontrolled hypertensive patients: rationale and study design of the SCRATCH-HTN study	A sham-controlled phase 2a trial (The SCRATCH-HTN) involving 63 participants is designed to test the safety, acceptability, and efficacy of transcutaneous autonomic neuromodulation for improving blood pressure control in patients with elevated blood pressure despite medication. If successful, this study might offer a novel non-invasive, inexpensive, self-administered device-based, neuromodulation strategy for patients with uncontrolled hypertension beyond conventional pharmacological management.	Study Protocol	42028232
Xinyu Zhang	Efficacy and safety of Shenqi Yangxin formula in patients with stable coronary artery disease based on CCTA: rationale, design, and study protocol for a randomized, double-blind, placebo-controlled study	A randomized, double-blind, placebo-controlled trial was designed to evaluate the efficacy and safety of Shenqi Yangxin Formula (SYF), a traditional Chinese medicine, in 60 patients with stable coronary artery disease, with primary end points assessed following12 weeks of treatment. This study is expected to provide novel evidence on the efficacy and safety of SYF in improving coronary hemodynamics and reducing residual inflammatory risk in patients with moderate coronary stenosis.	Study Protocol	42181649
Yanqing Wang	Early detection value of miR-29a in patients with acute myocardial infarction	In a prospective cohort study enrolling 52 patients with ST-elevation myocardial infarction and 39 healthy controls, serum miR-29a was found to be a novel biomarker for early detection of myocardial remodeling after acute myocardial infarction. By demonstrating that miR-29a is associated with ventricular remodeling and improves risk discrimination when combined with NT-proBNP and sST2, the findings support a more precise biomarker-based approach to post-infarction surveillance and heart failure risk stratification.	Original Research	42199207
Deng Pan	Efficacy and safety of FangJiHuangQi granule in patients with heart failure: a protocol of randomized, placebo-controlled trial	A randomized, double-blind,.placebo-controlled trial that recruits 100 patients with heart failure was designed to evaluate the efficacy and safety of FangJiHuangQi Granules (a Chinese medicine that alleviates heart failure), with the primary outcome—the changes of six-minute walk test assessed at week 24. This trial may provide complementary therapy in addition to conventional drug treatment and mechanistic insights into heart failure treatment.	Study Protocol	N.A.

One of the most rapidly evolving areas is the management of residual cardiovascular risk following acute coronary syndrome (ACS) and percutaneous coronary intervention (PCI). Although contemporary antithrombotic therapy, intensive lipid lowering, and early revascularization have significantly improved outcomes, persistent inflammation and endothelial dysfunction continue to drive recurrent events. The Mono Antiplatelet and Colchicine Therapy (MACT II) trial is designed to evaluate an aspirin-free, inflammation-guided strategy combining ticagrelor monotherapy with colchicine, with treatment duration tailored by high-sensitivity C-reactive protein levels. Similarly, the Xueshuantong injection trial protocol addresses myocardial injury and inflammatory risk in unstable angina using a biomarker-based assessment, reflecting growing interest in mechanism-oriented adjunctive therapies Wang et al.

Attention has also shifted from achieving large-vessel patency alone toward improving downstream myocardial perfusion. The slow flow/no-reflow phenomenon during percutaneous coronary intervention (PCI) for acute myocardial infarction (AMI) is a critical challenge. The pilot trial on electroacupuncture for slow flow/no-reflow provides preliminary evidence that this non-pharmacological intervention may improve coronary microvascular perfusion and reduce inflammation Wei et al.

Autonomic imbalance and lifestyle factors are increasingly recognized as key contributors to hypertension and adverse cardiovascular outcomes. The SCRATCH-HTN trial (A Sham-Controlled Randomised Trial evaluating the Safety, Acceptability and Efficacy of Autonomic neuromodulation in Uncontrolled Hypertensive patients) is designed to investigate a novel, non-invasive transcutaneous vagal sensory stimulation for uncontrolled hypertension Gupta et al., which may lead to development of device-based, self-administered therapies. Furthermore, the caffeine and sleep deprivation trial Mateo et al. demonstrates that acute caffeine intake during total sleep deprivation exacerbates blood pressure elevation and inflammatory responses, underscoring the importance of balancing performance-enhancing strategies with cardiovascular risk in sleep-restricted populations.

Precision cardiovascular medicine has accelerated the application of multi-omics and biomarker-guided risk stratification. The microRNA-29a (miR-29a) study Wang et al. demonstrates that this microRNA is a novel biomarker for early detection of myocardial remodeling after AMI and it improves early diagnosis when combined with other biomarkers [N-terminal pro-B-type natriuretic peptide (NT-proBNP), and Soluble suppression of tumorigenesis-2 (sST2)]. The multi-omics profiling study in elderly patients with CHD and mild cognitive impairment uncovers shared molecular pathways, supporting the emerging concept of cardio-cerebral interaction. The Shenqi Yangxin Formula trial uses coronary computed tomography angiography-derived parameters to evaluate the effect of the traditional Chinese medicine on plaque stabilization and residual risk in patients with stable coronary artery disease, embodying a precision-oriented, imaging-guided approach Zhang et al.

Finally, the scope of modern trials extends beyond coronary artery disease to chronic heart failure (HF), emphasizing functional capacity and quality of life. The FangJiHuangQi granule trial evaluates an adjunctive herbal therapy in HF patients using functional, biomarker, and patient-reported outcomes, reflecting a more comprehensive, patient-centered management paradigm. Complementing this, the ginseng berry saponins trial in stable coronary artery disease Ge et al. focuses on improving cardiorespiratory fitness, a key determinant of long-term prognosis. In addition, similar cardiovascular safety profile and potassium lowering effects were detected in hyperkalemic patients between levalbuterol and albuterol, two bronchodilator medications for pulmonary disease Shebli et al.

Collectively, these contemporary cardiovascular clinical studies address residual cardiovascular risk beyond current standard treatments by assessing traditional medicine, inflammation-targeted therapies and autonomic modulation, and uncover novel biomarkers and molecular signature. Together, they provide valuable insights that may inform new drug discovery and future strategies for individualized prevention and long-term cardiovascular disease management.
